# The Autotaxin-LPA Axis Emerges as a Novel Regulator of Smooth Muscle Cell Phenotypic Modulation during Intimal Hyperplasia

**DOI:** 10.3390/ijms24032913

**Published:** 2023-02-02

**Authors:** Utsab Subedi, Shrivats Manikandan, Susmita Bhattarai, Papori Sharma, Sudha Sharma, Hong Sun, Sumitra Miriyala, Manikandan Panchatcharam

**Affiliations:** Department of Cellular Biology and Anatomy, Louisiana State University Health Sciences-Shreveport, Shreveport, LA 71130, USA

**Keywords:** lysophosphatidic acid, autotaxin, neointimal hyperplasia, restenosis, smooth muscle cells

## Abstract

Neointimal hyperplasia is characterized by a loss of the contractile phenotype of vascular smooth muscle cells (VSMCs). Our group has recently shown that VSMC proliferation and migration are mediated by lysophosphatidic acid (LPA) during restenosis, but the role of autotaxin (ATX; lysophospholipase D), which produces LPA, remains unclear. Endothelial denudation of the mouse carotid artery was performed to induce neointimal hyperplasia, and the extent of damage caused by the ATX-LPA axis was assessed in VSMCs. We observed the upregulation of ATX activity (*p* < 0.0002) in the injured carotid artery using an AR2 probe fluorescence assay. Further, the tissue carotid LPA levels were elevated 2.7-fold in carotid vessels, augmenting neointimal hyperplasia. We used an electrical cell–substrate impedance sensor (ECIS) to measure VSMC proliferation and migration. Treatment with an ATX inhibitor (PF8380) or LPA receptor inhibitor (Ki16425) attenuated VSMC proliferation (extracellular signal-regulated kinases) activity and migration in response to recombinant ATX. Indeed, PF8380 treatment rescued the aggravated post-wire injury neointima formation of carotid arteries. The upregulation of ATX following vessel injury leads to LPA production in VSMCs, favoring restenosis. Our observations suggest that inhibition of the ATX-LPA axis could be therapeutically targeted in restenosis to minimize VSMC phenotypic modulation and inflammation after vascular injury.

## 1. Introduction

Endovascular interventions such as balloon angioplasty and stent placement are the first line of therapy for treating atherosclerosis and thrombosis [1]. These procedures play a vital role in restoring blood flow to the target organs, such as the heart and brain [2]. However, regardless of the potential benefits of restoring blood flow, these procedures are limited due to restenosis characterized by neointimal hyperplasia. Almost sixty percent of patients develop restenosis within twelve months of angioplasty [3], and the number grows further with diabetes [4]. Neointimal hyperplasia is a post-pathological event that occurs due to the proliferation and migration of vascular smooth muscle cells (VSMCs) from the medial region to the intimal area in the artery. This intervention leads to obstruction in blood flow in the blood vessels, such as in the heart, causing myocardial infarction, and in the brain, causing stroke [5]. This study focuses on the role of the autotaxin (ATX)-lysophosphatidic axis in neointimal hyperplasia following vascular injury. Lysophosphatidic acid (LPA) is a highly potent bioactive phospholipid found in various isoforms and functions through its G-coupled receptors (LPAR1-6), leading to significant downstream signaling. The receptor for the advanced glycation end product (RAGE) has recently been found to be a potent receptor of LPA [6]. LPA can exert various cellular responses across multiple cell types, including proliferation, migration, survival, platelet aggregation, VSMC contraction, and chemokine and cytokine secretion [7,8]. LPA is linked with many diseases, including lung fibrosis, cancers, inflammation, cardiovascular disease, and neurological conditions [9,10]. Almost every type of vascular cell, including vascular endothelial cells, VSMCs, fibroblasts, T-lymphocytes, platelets, and monocytes, responds to LPA [11], leading to angiogenesis, vasculogenesis, and vascular remodeling [12,13,14,15]. LPA and its receptors are responsible for various vascular pathologies. LPA is an active component of atherosclerosis plaque and induces endothelial cell and platelet activation [16,17]. Previous studies have shown that LPAR1 and LPAR2 [18] or LPAR1 and LPAR3 [19] are involved in neointimal hyperplasia following vascular injury. Neointimal hyperplasia is inevitable following vascular injury, and LPA receptor-mediated signaling varies with co-morbidity. A recent study has shown that LPA receptor-mediated signaling may play a role in the proliferation and migration of VSMCs during experimental atherosclerosis [20].

LPA is extensively generated in the body from phospholipids involving the glycoprotein enzyme ATX. Lysophosphatidylcholine is highly abundant in plasma as a precursor of LPA production. Global inactivation of the enzyme ATX leads to embryonic lethality due to a lack of vascular [21] and nervous system development [22], but it is dispensable in adult life [23]. The role of ATX has been studied in various pathologies, such as stroke [24,25], cancer [26], lung fibrosis [27], liver fibrosis [28], and rheumatoid arthritis [29]. Activated platelets release ATX stored in alpha granules [30]. Activated platelet β3 integrins promote LPA production and LPA-dependent response by binding with ATX [31].

Furthermore, LPA enhances platelet fibronectin binding during the tissue repair process following vascular injury, resulting in matrix deposition [32]. As ATX is a significant player in producing LPA, earlier evidence indicates that it could be used as a strong therapeutic target in cardiovascular disease due to its involvement in physiological and pathological functions [33]. During vascular injury, oxidative stress leads to LPA production, and its signaling causes the VSMCs to proliferate and migrate to form neointimal hyperplasia. However, even though LPA is involved in neointimal hyperplasia, the role of ATX in neointimal hyperplasia is still unexplored. Therefore, this study focuses on the effect of the ATX-LPA axis on neointimal hyperplasia following vascular injury. 

## 2. Results

### 2.1. Mouse Model for Endothelial Denudation

**Figure 1:**
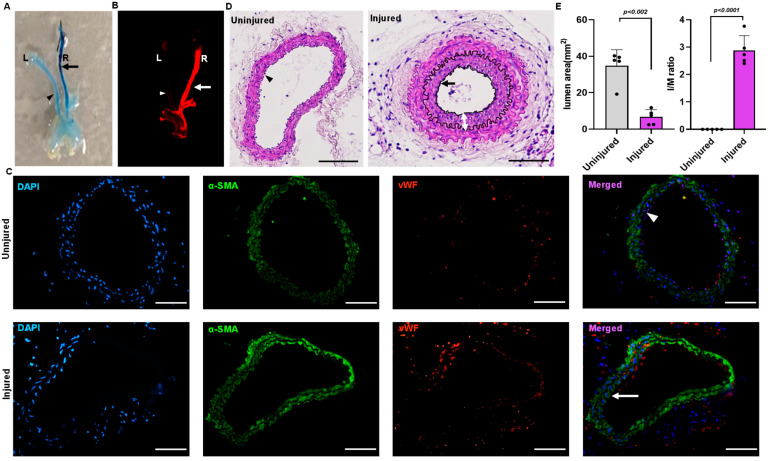
Mouse model of endothelial denudation. (**A**) Evans blue staining of right injured carotid artery and left uninjured carotid artery at day 1. (**B**) Infrared imaging of the right injured carotid artery and left uninjured carotid artery at day 1 using the LI-COR Odyssey machine, with Evans blue fluorescence measured at 700 nm. (**C**) Immunofluorescence staining of the sections dissected from the injured and uninjured carotid artery at day 1. Scale bar = 50 μm. (**D**) Hematoxylin and eosin staining of the uninjured and injured carotid artery 4 weeks after surgery. Scale bars, 50 μm. (**E**) Quantification of lumen area and IM ratio. The arrow indicate the injured artery, and the arrowhead indicates the uninjured artery. The double arrow indicates the thickness of the neointima. All values are mean ± SD (*n* = 5) compared to the uninjured vessel using the Mann–Whitney U test.

Endothelial denudation injuries was performed at the right carotid artery in three-month-old C57BL/6J mice. Evans blue staining of the carotid artery of the mice that underwent endothelial denudation injuries showed dark blue coloration compared to the uninjured arteries (Figure 1A). Carotid image analysis using an infrared imager (LI-COR Odyssey), wherein Evan blue provides autofluorescence at 700 nm, also showed a prominent Evans blue infiltration in the injured right carotid vascular wall following endothelial denudation (Figure 1B). In addition, immunostaining for the endothelial-specific marker von Willebrand factor (vWF) and the VSMC-specific marker α-actin was performed to further confirm vascular endothelial denudation. The finding indicates the absence of endothelial cells in the injured right carotid artery compared to the uninjured left carotid artery (Figure 1C). The carotid artery was harvested, paraffin-embedded, and sectioned on day 28. The uninjured (left carotid) artery was compared to the injured (right carotid) artery for neointimal hyperplasia through staining with hematoxylin and eosin. The neointimal hyperplasia was quantified using Image J software and represented as intima by media ratio and luminal area. The hematoxylin and eosin staining showed an increase in neointimal hyperplasia (*p* < 0.002) with decreased luminal size (*p* < 0.001) following vascular injury in the endothelial denudated vessel as compared to uninjured control at day 28 (Figure 1D,E).

### 2.2. Elevation of ATX Activity and Expression Following Endothelial Denudation Injury

**Figure 2:**
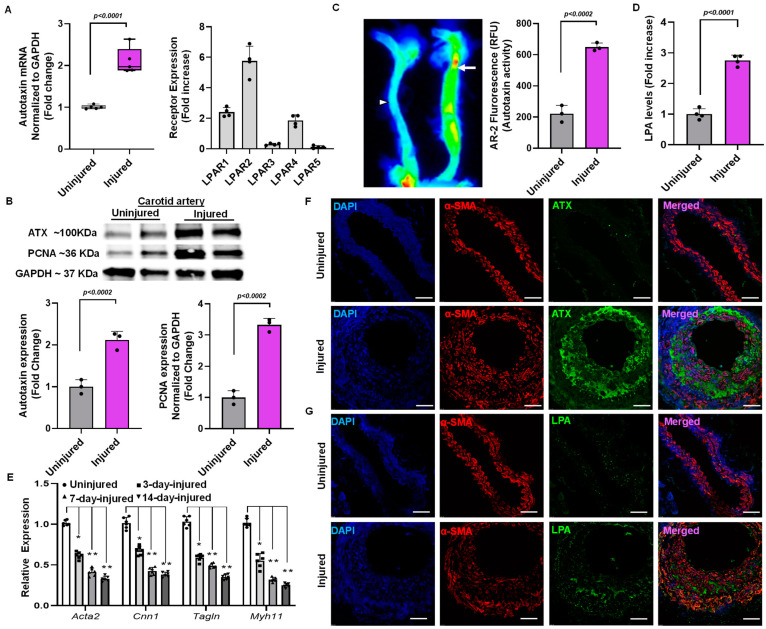
Vascular injury alters ATX expression in mouse carotid arteries. (**A**) ATX and LPA receptor mRNA expression was measured from the injured and uninjured arteries on day 14. (**B**) Immunoblotting for ATX and PCNA following 14 days of vascular injury. (**C**) The ATX enzymatic activity test was measured with AR2 fluorescence and quantified in terms of relative fluorescence units (RFUs) in uninjured and injured mouse carotid arteries. The arrow indicate the injured artery, and the arrowhead indicates the uninjured artery (**D**) The ELISA technique was used to measure LPA levels 14 days after vascular injury. All values are mean ± SD (*n* = 3) compared to the uninjured vessel using the Mann–Whitney U test. (**E**) Quantitative polymerase chain reactions were used to measure the expression of the SMC differentiation markers *Acta2* (SM-actin), *Cnn1* (calponin), *Tagln* (SM22), and *Myh11* (SM-myosin heavy chain) at different time points. The data were presented as the mean ± SD and analyzed using a one-way ANOVA followed by Tukey’s test for post hoc comparison. *n* = 6 for each group, * *p* < 0.05, and ** *p* < 0.001. (**F**) Immunohistochemistry for ATX and smooth muscle actin (α-SMA) after vascular injury at day 14. Scale bar = 10 μm. (**G**) Immunohistochemistry for LPA and smooth muscle actin after vascular injury at day 14. Scale bar = 10 μm. LPA—lysophosphatidic acid; LPAR—lysophosphatidic acid receptor; ATX—autotaxin; PCNA—proliferating cell nuclear antigen.

After surgery, the carotid arteries were collected for a reverse transcription polymerase chain reaction on day 14. The injured endothelial denudated carotid artery showed more than a two-fold increase in ATX mRNA level compared to an uninjured carotid artery at day 14 (Figure 2A). In addition, the injured carotid arteries exhibited more than a two-fold increase in the mRNA expression of various LPA receptors (Figure 2A). The uninjured and injured carotid artery tissues collected were blotted for ATX and proliferating cell nuclear antigen (PCNA) on day 14. ATX (*p* < 0.002) and PCNA (*p* < 0.002) protein expression was significantly upregulated in injured arteries compared to uninjured arteries when normalized to GAPDH (Figure 2B). After establishing upregulation of ATX after vessel injury, we investigated whether ATX enzymatic activity is regulated in the production of LPA. An ATX activity assay was performed using an AR2 fluorescence assay after vascular injury on day 14. Figure 2C shows that ATX activity was significantly upregulated in the injured carotid artery compared to the uninjured artery on day 14. The AR2 fluorescence measured using the LI-COR Odyssey infrared imager directly correlates with ATX activity in the carotid arteries in real time. Following the observation of increased ATX mRNA and protein expression and enhanced ATX activity in the injured vessels, we analyzed tissue LPA after injury. After vascular injury, we found more than a three-fold increase in tissue LPA levels compared to uninjured carotid arteries (Figure 2D) on day 14. Furthermore, the contractile markers *Acta2, Cnn1, Tgln,* and *Myh11* were decreased in the injured carotid arteries compared to uninjured carotid arteries (Figure 2E), as indicated by assessment at days 3, 7, and 14 after wire injury/sham procedure. To further substantiate the role of the ATX-LPA axis in VSMCs mediating neointimal hyperplasia, we performed immunohistochemical analysis for ATX, α-smooth muscle actin, and LPA (Figure 2F,G). Our findings indicate significant upregulation of ATX and LPA levels following a vascular injury on day 14. Our confocal microscopy results suggest the co-localization of ATX and LPA to VSMC neointimal areas, which are highly upregulated and expressed following vascular remodeling.

### 2.3. ATX Inhibition Decreased VSMC Proliferation and Migration

**Figure 3:**
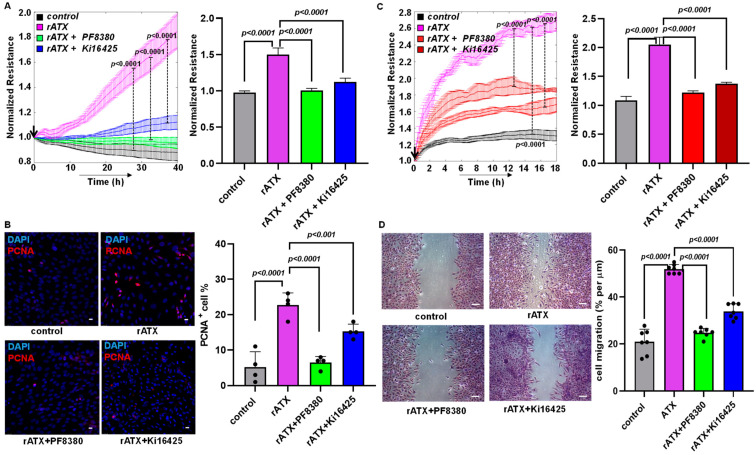
ATX alters VSMC proliferation and migration. (**A**) Measurement of cell proliferation using ECIS in mouse VSMCs with control (no rATX), rATX (10 nM), PF8380 (10 μM), or Ki16425 (10 μM) treatment. All values are expressed as mean ± SD (*n* = 4) with *p*-values calculated by one-way ANOVA. (**B**) Immunocytochemistry for PCNA in mouse VSMCs with control (no rATX), rATX (10 nM), PF8380 (10 μM), or Ki16425 (10 μM) treatments and quantified PCNA-positive cell percentage obtained by dividing PCNA-positive cells by the total number of cells. Scale bar = 25 μm. All values are expressed as mean ± SD (*n* = 4) with *p*-values calculated by one-way ANOVA. (**C**) Measurement of cell migration using ECIS in mouse VSMCs with control (no rATX), rATX (10 nM), PF8380 (10 μM), or Ki16425 (10 μM) treatments. All values are expressed as mean ± SD (*n* = 4) with *p*-values calculated by one-way ANOVA. (**D**) Wound healing scratch assay was performed to measure cellular migration in mouse VSMCs with control (no rATX), rATX (10 nM), PF8380 (10 μM), or Ki16425 (10 μM) treatments. Scale bar = 100 μm. All values are expressed as mean ± SD (*n* = 4) with *p*-values calculated by one-way ANOVA. rATX—recombinant active ATX; PF8380—ATX inhibitor; Ki16425—LPAR1-3 inhibitor; PCNA—proliferating cell nuclear antigen.

Previously, VSMC proliferation and migration have been observed to contribute to neointimal hyperplasia [34]. Furthermore, our earlier findings indicate that LPA treatment increases VSMC proliferation [35], and migration is one of the key factors contributing to neointimal hyperplasia [18,34,36]. Even though LPA was shown to regulate restenosis [18,34,37] significantly, ATX’s role in vascular remodeling and intimal hyperplasia remains unexplored. Therefore, we delineated the effect of ATX using isolated, cultured VSMCs from the murine aorta. VSMC proliferation and migration were determined in real time using an ECIS machine, which was followed by immunocytochemistry. We tested the effects of recombinant active ATX with and without PF8380 (ATX inhibitor) or LPAR1/LPAR2/LPAR3 receptor inhibitor Ki16425 treatment in our in vitro cell culture model. A profound increase in VSMC proliferation upon exposure to recombinant active ATX treatment was measured in real time using ECIS. Furthermore, a robust increase in proliferation upon exposure to recombinant active ATX treatment was confirmed by immunocytochemistry staining for proliferating cell nuclear antigen (PCNA; proliferation marker) at 48 h (Figure 3B). Both PF8380 and Ki16425 significantly reduced the effect of recombinant ATX-mediated VSMC proliferation as measured through the ECIS assay and the positive PCNA staining of VSMCs by immunocytochemistry. Next, we examined VSMC migration towards recombinant active ATX with or without PF8380 or Ki16425 (Figure 3C,D). We observed a two-fold increase in resistance by ECIS on VSMC migration upon exposure to recombinant active ATX. The migratory effect of VSMCs was further confirmed using a traditional wound healing scratch assay towards recombinant active ATX, which showed a more than a two-fold increase. With PF8380 and Ki16425 treatment, the migratory response of VSMCs mediated by recombinant active ATX was highly attenuated, as observed with both ECIS and the wound healing scratch assay. Together, these results indicate that the ATX-LPA axis regulates the proliferation and migration of VSMCs, particularly under pathological conditions where ATX is upregulated after vascular injury. These results suggest that the ATX-LPA axis can trigger VSMC proliferation and migration.

### 2.4. ATX-Mediated Phosphorylation of AKT–ERK Signaling in VSMCs

**Figure 4:**
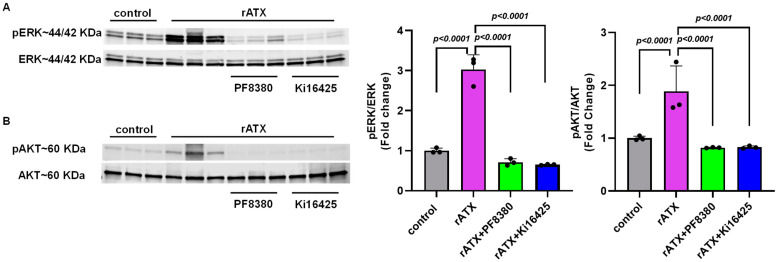
AKT/ERK signaling in VSMCs induced by ATX. (**A**) Western blot analysis of phosphorylated ERK was performed in mouse VSMCs with control (no rATX), rATX (10 nM), PF8380 (10 μM), or Ki16425 (10 μM) treatment. (**B**) Western blot analysis of phosphorylated AKT was performed in mouse VSMCs with control (no rATX), rATX (10 nM), PF8380 (10 μM), or Ki16425 (10 μM) treatment. All values are expressed as mean ± SD (*n* = 3) with *p*-values calculated by one-way ANOVA. rATX-recombinant active autotaxin; PF8380—autotaxin inhibitor; Ki16425—LPAR1-3 inhibitor.

To investigate the mechanism(s) by which ATX might regulate vascular injury responses, we measured cell proliferation signaling markers for these events after exposure of VSMCs to recombinant active ATX. To identify whether the AKT–ERK signaling pathway is associated with the proliferation and migration of VSMCs induced by ATX, we measured the phosphorylation of these proteins through Western blotting. We found that VSMC exposure with recombinant active ATX (10 nmol/L) increased ERK activation, as measured by the ratio of phospho-ERK to total ERK at 2 h. Furthermore, we observed that the ERK activity induced by recombinant active ATX was significantly reduced by treatment with the ATX inhibitor (PF8380; *p* < 0.0001) or with the LPA receptor inhibitor (Ki16425; *p* < 0.0001) (Figure 4A). Similarly, we observed that phospho-AKT levels were significantly elevated compared to the control group when the VSMCs were exposed to recombinant active ATX (10 nmol/L) for 2 h. In addition, AKT activity induced by recombinant ATX was reduced by PF8380 and Ki16425 by ~50% (Figure 4B). These results indicate that the ATX-LPA axis mediates VSMC proliferation and migration through the AKT/ERK signaling pathway, causing vascular remodeling.

### 2.5. ATX Inhibitor Reduces Inflammation Following Injury

**Figure 5:**
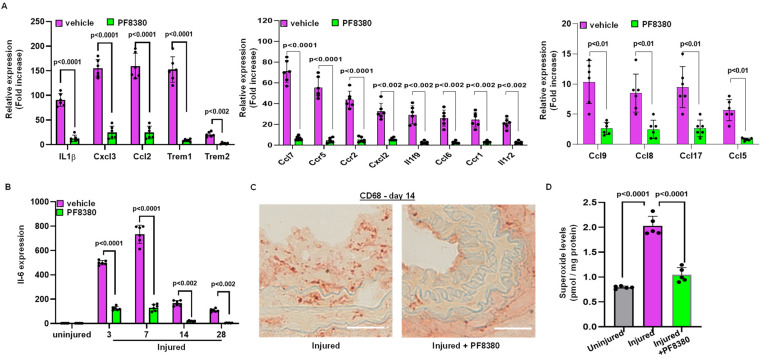
Treatment with the ATX inhibitor rescues mRNA encoding proteins associated with inflammation following injury. (**A**) Chemokine and cytokine mRNA expression was measured on day 3 with and without PF8380 treatment following injury. (**B**) Transcriptional mRNA expression levels of IL-6 with and without PF8380 treatment for different time points. (**C**) Representative sections of mouse carotid artery stained with CD68 antibody (C68-target for macrophages) with and without PF8380 treatment at 2 weeks after injury. Scale bar = 50 μm. (**D**) Superoxide measured using high-performance liquid chromatography in uninjured, injured, and PF8380-treated mice at day 14. All values are mean ± SD (*n* = 6) compared to the uninjured vessel using the Mann–Whitney U test.

We further identified the source of inflammation. Following carotid artery denudation injury, we observed increased mRNA expression of chemokines and cytokines in the injured carotid artery on day 3, including mRNAs such as interleukin-1β (IL1 β), interleukin-6 (IL6), and chemokine ligand 2 (Ccl2, also known as monocyte chemoattractant protein 1 or MCP1). We also measured IL6 mRNA expression at different time points following injury, both with and without PF8380 treatment. Our finding suggests that mRNA expression of cytokines and chemokines was abrogated with ATX inhibitor PF8380 treatment (Figure 5A,B). Furthermore, immunostaining with anti-CD68 antibodies confirmed macrophage infiltration upon vascular injury, wherein PF8380 treatment abolished macrophage infiltration, as observed on day 14 following injury (Figure 5C). LPA-mediated oxidative stress was assessed on day 14, and it was observed that the increased superoxide (O_2_^•−^) levels, as measured using high-performance liquid chromatography in the injured carotid artery, were inhibited considerably (*p* < 0.0001) with PF8380 treatment (Figure 5D). 

### 2.6. ATX Inhibitor Reduces Neointimal Hyperplasia

**Figure 6:**
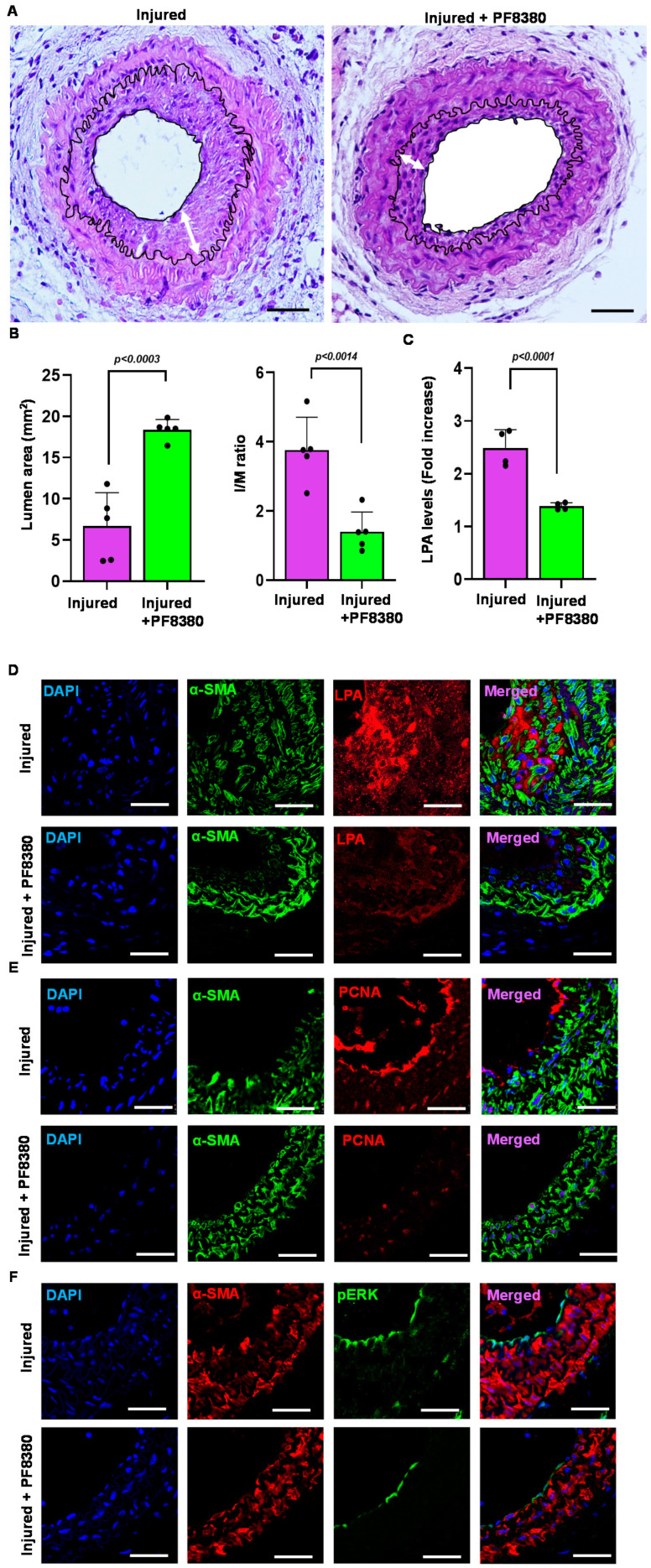
Treatment with the ATX inhibitor rescues neointimal hyperplasia. (**A**) Hematoxylin and eosin staining sections of the injured carotid artery with and without PF8380 treatment at 4 weeks after injury. Scale bar = 50 μm. (**B**) Quantifying luminal area and I/M ratio with and without PF8380 treatment at 4 weeks after injury. (**C**) Carotid tissue LPA levels with and without PF8380 at 3 weeks after injury. (**D**) Mouse carotid artery stained for LPA with and without PF8380 treatment at 4 weeks after vascular injury. Scale bar = 10 μm. (**E**) Immunofluorescence staining for PCNA in injured carotid arteries with and without PF8380 treatment at 4 weeks after vascular injury. Scale bar = 10 μm. (**F**) Immunohistochemistry for phospho-ERK in injured carotid arteries with and without PF8380 treatment at 4 weeks after vascular injury. Scale bar = 10 μm. All values are mean ± SD (*n* = 3) compared to the uninjured vessel using the Mann–Whitney U test. LPA—lysophosphatidic acid; LPAR—lysophosphatidic acid receptor; ATX—autotaxin; PCNA—proliferating cell nuclear antigen.

Following carotid artery denudation injury, we observed a significant increase in vessel-associated ATX mRNA expression, protein levels, and activity compared to uninjured vessels at day 14 (Figure 2A–C). To determine the molecular underpinnings of the enhanced injury response, we examined the consequences of ATX inhibition in more detail by using ATX inhibitor PF8380 in our restenosis model. PF8380 was administered for 28 days following vascular injury. The carotid vessels were isolated and analyzed after 28 days of injury. Consistent with VSMCs serving as a major source of vessel-associated ATX, the PF8380 inhibitor attenuated ATX-induced neointimal hyperplasia four weeks after the injury, as observed through a significant reduction in the intima/media ratio (Figure 6A,B). PF8380 inhibitor treatment enhanced the luminal area (Figure 6A,B); however, the media thickness remained unchanged. ATX was thus identified as the source of LPA production following vascular injury due to downstream signaling being triggered. ATX inhibitor PF8380 treatment showed a robust decrease in carotid tissue LPA level at 21 days of injury as measured by the ELISA technique (Figure 6C) compared to the injured artery without treatment. We observed significant upregulation of LPA, phospho-ERK, and PCNA expression in vivo and in vitro; thus, we used immunohistochemical analysis and probed for PCNA, LPA, and phospho-ERK at 28 days of vascular injury with and without PF8380 treatment. Our data suggest a significant decrease in PCNA, LPA, and phospho-ERK expression following PF8380 treatment (Figure 6D–F). On day 21 after injury, we observed that the increased carotid tissue LPA levels were significantly inhibited by ATX inhibitor PF8380 treatment (*p* < 0.0001). Together, these observations demonstrate a role for the ATX-LPA axis in enhancing the proliferative and migratory responses of VSMCs causing the neointima, and PF8380 treatment can protect the vasculature and mitigate the adverse responses mediated by ATX after vascular injury. 

## 3. Discussion

Neointimal hyperplasia leading to restenosis is one of the major complications of endovascular surgeries [37,38], due to which surgeons have faced a significant challenge in controlling post-interventional vascular complications. VSMCs are the primary type of cells that maintain vessel diameter, vascular tone, and vascular homeostasis [39] and are mainly involved in cellular proliferation and migration during neointimal hyperplasia [40]. In addition, endothelial cells maintain the barrier function controlling vascular permeability [41]. During the endovascular intervention, the endothelial layer of the vascular wall gets denudated or destroyed, exposing VSMCs to the hemodynamic environment and triggering VSMC proliferation and migration. Therefore, targeting the modulation of VSMC phenotypes after endovascular surgeries has been a critical therapeutic target in terms of restricting neointimal hyperplasia [42,43]. This study addresses the role of the ATX-LPA axis in a clinically relevant mouse restenosis model through endothelial denudation of the carotid vasculature for the first time. In our injury model, we assessed the lack of endothelial lining after vessel injury that was accompanied by neointimal hyperplasia of VSMCs causing vessel narrowing. The levels of ATX, the precursor enzyme for LPA production, were observed to be increased by more than two-fold at the mRNA and protein levels. Enhanced ATX enzymatic activity might ramp up the conversion of the substrate lysophosphatidylcholine and produce more LPA in pathological settings. Thus, we performed and analyzed ATX activity in mouse carotid vessels using an AR2 fluorescence probe in real time. We observed a three-fold increase in ATX enzymatic activity in the injured artery. We next investigated whether ATX could be produced locally at the sight of injury or if it is recruited from circulation. To address the source of ATX, we performed co-immunostaining using a VSMC marker (α-actin) and probed for ATX and LPA. These findings were further confirmed by measuring carotid tissue LPA levels with an ELISA assay. Our finding suggests that the ATX-LPA axis is highly regulated in the injured vessels and is more confined to VSMCs. Consistent with VSMCs serving as a major source of vessel-associated ATX, we observed that elevated tissue LPA levels elicit downstream signaling through its G-protein-coupled receptors. LPA is a potent mitogen that mediates cellular proliferation and migration in various cell types [44,45,46] and cancerous cells [42,45,47] via its receptors. When we assessed the mRNA expression levels of different LPA receptors after injury, we observed a two- to six-fold increase in LPAR1 and LPAR2 receptor expression levels, consistent with our earlier reports [18]. Even though we observed that most of the LPA receptor expression levels were modulated after vascular injury, our earlier findings with specific LPA receptor knockout mice subjected to experimental restenosis suggest the prominent role played only by LPAR1-3 in VSMCs, namely the mediation of neointimal hyperplasia [18].

After an injury, VSMCs in the medial region of the intimal layer undergo proliferation and migration, thus resulting in neointimal hyperplasia and vascular remodeling. LPA is a potent bioactive lipid that induces VSMC proliferation and migration, contributing to neointimal hyperplasia [48,49,50]. To assess the role of the ATX-LPA axis on VSMC cellular proliferation and migration, we exposed the VSMCs to recombinant active ATX with and without the ATX inhibitor (PF8380) or LPA receptor inhibitor (Ki16425) and measured resistance using ECIS. Specifically, treatment with PF8380 or Ki16425 could abolish the ATX-LPA axis-mediated proliferation and migration of VSMCs, as observed in the decreased resistance shown in ECIS tests between the groups. Furthermore, LPAR receptor inhibition has shown some beneficial effects in inflammation [51], cancer [52,53], and various cardiovascular diseases [33,46]. These results suggest that ATX activity enhances VSMC proliferation and migration, thus leading to vascular remodeling. Therefore, the adverse effects of ATX on VSMCs can be curtailed with ATX inhibitors or through the inhibition of LPA receptor-mediated signaling. These results indicate that ATX-derived LPA enhances the proliferative and migratory behavior of VSMCs through its LPAR-mediated signaling after injury.

To further investigate the role of the ATX-LPA axis on carotid vascular remodeling after injury in vivo, the mice were treated with ATX inhibitor PF8380 daily for four weeks. First, we measured the carotid tissue LPA levels through an ELISA assay and found that PF8380 treatment could significantly reduce ATX-induced LPA after injury. Immunohistochemical analyses were performed using LPA antibodies on these carotid sections to further complement our findings. We observed a significant reduction in LPA levels by immunostaining these injured carotid vessels with PF8380 treatment on day 28. Next, we used PCNA, a proliferation marker, to track the status of these VSMCs after injury. Our data suggest that PCNA was significantly reduced with PF8380 treatment in the injured vessels, suggesting that inhibition of ATX activity might rescue LPA-induced vascular remodeling after injury. Our results align with earlier findings that LPA mediates its signaling through activated ERK and AKT pathways in VSMCs, leading to neointimal hyperplasia [18,35,54,55,56,57]. Furthermore, ERK and AKT activity abrogation in cell culture mitigated VSMC proliferation and migration [18,34,35,58]. We observed a significant increase in ERK (*p* < 0.0001) and AKT (*p* < 0.0001) activity in the VSMCs treated with recombinant active ATX. Treatment with PF8380 or Ki16425 could abolish ERK and AKT activity in VSMCs in the presence of recombinant active ATX. Immunohistochemical analysis of the injured vessels showed enhanced phospho-ERK staining in the neointima, and PF8380 treatment could abrogate these signaling responses in the VSMC on day 28. Our results indicate that PF8380 treatment can limit restenosis by decreasing neointima formation and maintaining luminal size after vascular injury. These results suggest that ATX inhibition might rescue vascular remodeling after injury by reducing the vasculature’s LPA-mediated signaling response. Furthermore, PF8380 was recently shown to protect against intestinal fibrostenosis by decreasing the proliferation and differentiation of intestinal fibroblasts mediated by ATX [59].

A study conducted by Yu et al. and others has shown that LPA increases proinflammatory cytokines such as IL-6, IL-8 and growth factors in various cancers [26,60]. Earlier, we reported that deletion of the LPA dephosphorylating enzyme (*PLPP3*) in the VSMCs led to elevated IL-6 levels due to increased LPA levels in the vasculature [35]. We observed a marked increase in local inflammatory markers in our mouse carotid denudation model at an earlier time point (day 3). Based on our observation, various chemokines and cytokines are abrogated to almost the basal level upon PF8380 treatment. This suggests that infiltration of chemokines and cytokines is limited in the earlier stages of vascular surgery upon PF8380 treatment, thus limiting inflammatory response in the artery. We observed that mRNA expression levels of IL6 were upregulated after injury in a time-dependent manner. At an earlier time, up to 7 days after injury, we observed that the IL6 expression is significantly upregulated compared to the later stages in our mice injury model, suggesting the role of inflammatory cells in vascular remodeling. The enhanced inflammatory response after injury was not observed with the PF8380 treatment. In the earlier part of an inflammatory process, infiltrated monocytes inside the injured vascular wall differentiate into macrophages, which release various growth factors that stimulate VSMCs proliferation and migration and leads to restenosis. We observed more macrophage infiltration in the neointimal area on day 14 by immunostaining for CD68 after injury.

In contrast, PF8380 treatment on the injured carotid artery vessels showed a smaller neointimal area with very few macrophage infiltrations on day 14. These results signify the role of the ATX-LPA axis in the inflammatory process after vessel injury. Furthermore, ATX-LPA-mediated oxidative stress was assessed, as measured by increased superoxide (O_2_^•−^) levels in the injured carotid vessels. Treatment with PF8380 was able to partially rescue oxidative stress mediated by ATX after injury by ~70%.

Therefore, these results suggest that increased ATX expression in VSMCs after vascular injury could enhance the production of LPA. LPA might stimulate the VSMCs through its G-protein-coupled receptors, causing proliferation and migration and leading to neointimal hyperplasia [18,48,49,61]. Our observation shows that PF8380 treatment in VSMCs abolishes ATX-LPA axis-mediated ERK/AKT activity. Furthermore, enhanced VSMC proliferation and migration mediated by the ATX-LPA axis was mitigated with an ATX inhibitor or LPA receptor inhibitor. Overall, PF8380 treatment rescued vascular remodeling in mice from enhanced neointimal hyperplasia mediated by the ATX-LPA signaling nexus after injury. Our results suggest that targeting the ATX-LPA axis can be a safer therapeutic approach to preventing neointimal hyperplasia in patients undergoing balloon angioplasty or stent replacement. 

## 4. Methods and Materials

### 4.1. Animal Model of Vascular Injury

All the procedures and protocols for animal studies were approved by the Institutional Animal Care and Use Committee (IACUC) at LSU Health Sciences Center–Shreveport. The protocols were developed in accordance with the NIH Guide for Care and Use of Laboratory Animals. A carotid artery wire injury model, or sham, was performed in three-month-old C57BL/6J mice. The left carotid artery was exposed through a midline incision in the neck of approximately 1 cm. The entire length of the left carotid artery was exposed, and the artery was obstructed with a slip knot immediately proximal to the point of bifurcation using a 7-0 silk suture (or a vascular clamp). Another 7-0 slip knot suture was placed around the common carotid artery immediately distal from the branch point of the external carotid. A transverse arteriotomy was made between the 7-0 sutures, and the resin probe was inserted and advanced towards the aorta arch and withdrawn five times. The probe was removed, and the proximal 7-0 suture was ligated. Isoflurane (induction at 3% and maintenance at 1.5%) was used to anesthetize mice and was mixed with 30% O_2_/70% N_2_ gas via a facemask. A temperature-controlled heating pad (Harvard Apparatus, March, Germany) was used to maintain the temperature of mice at 37 °C throughout the surgery. Once restoration of blood flow through the carotid branch points was confirmed, the incision was closed with 6-0 sterile surgical silk. The sham group only had the left carotid artery exposure performed, not the injury. The entire procedure was performed within 30 min. Mice were given carprofen (2 to 5 mg/kg) postoperatively by subcutaneous injection. Animals were allowed to recover in a cage warmed to 37 °C. Soft food and clean bedding were in place for the mice. The mice were euthanized at selected time points between 1 h and four weeks after the procedure to assess vascular remodeling response. Hematoxylin and eosin staining were performed to measure the neointimal hyperplasia. Mice were injected intraperitoneally with Evans blue (1.2 mL/kg, 0.1 mL of a 1% solution in PBS) 3 h before isolating the carotid artery following the surgery to confirm endothelial denudation. Fluorescence intensity was measured and quantified at 700 nm in the LI-COR Odyssey (LI-COR Biosciences, Lincoln, NE, USA) near-infrared imager (NIR).

### 4.2. Cell Culture

Thoracic aorta from three C57BL/6J mice < 10 weeks old were isolated under the dissection microscope. The aortas were kept in a solution containing HBSS (with calcium and magnesium) and collagenase type II (Worthington Biochemical, Lakewood, NJ, USA; 175 units/mL, LS004174) and agitated at 37 °C for 30 min. The adventitial layer was removed under a surgical microscope. The inner layer of the artery (endothelial layer) was scraped out with a sterile cotton swap. Later, the aortas underwent sequential digestion with collagenase type II and elastase (Sigma-Aldrich, St. Louis, MO, USA; 0.5 mg/mL), which yielded 100,000 cells per aorta. Purified VSMCs were grown in DMEM media containing 0.5 ng/mL EGF, 5 µg/mL insulin, 2 ng/mL β-FGF, 10% FBS, 100 units/mL penicillin, and 100 µg/mL streptomycin [18,35]. VSMC lineage was confirmed by immunostaining for α-actin (Sigma) in >99% of the cells. Experiments involving VSMCs were performed using cells with a passage number ≤5. The cells were maintained at 37 °C in a humidified atmosphere of 5% CO_2_.

### 4.3. ATX Activity Assay

ATX activity was measured and quantified in carotid arteries 7 and 14 days following vascular injury using a fluorogenic analog of LPC (AR2 probe) as previously described [24,25]. Briefly, AR2 (0.5 mg/kg) was injected retro-orbitally 3 h before isolating the carotid artery for imaging. The mice were then perfused transcardinally to remove blood from the vessels. Finally, the vessels were imaged and quantified using the LI-COR Odyssey (LI-COR, Biosciences, Lincoln, NE, USA) near-infrared imager at 800 nm. 

### 4.4. Migration and Proliferation Assay Using an Electric Cell–Substrate Impedance Sensor (ECIS)

The migration wound healing assay was performed using the ECIS ΖΘ machine (Applied Biophysics, Troy, NY, USA) [62]. Mouse VSMCs were seeded at 80,000 per well into the gold-plated electrode chamber of the ECIS array (Applied Biophysics; 8WIE) and allowed to grow until a confluent monolayer was formed. Next, the cells were starved for 72 h. Following 72 h starvation, electrical wounds were made in the ECIS plate by elevating the voltage pulse to a 40 kHz frequency at a 3.5 V amplitude for 30 s. This sudden elevation in voltage causes cell death and detachment from the active electrode. The medium was then aspirated and treated with 10 nM of recombinant active ATX (#E-4000, Echelon Biosciences, Salt Lake City, UT, USA) with or without 10 µM of ATX inhibitor PF8380 (#B-0702, Echelon Biosciences, Salt Lake City, UT, USA) [24,25], or an LPA receptor antagonist (#B-0730, Echelon Biosciences, Salt Lake City, UT, USA) [62]. The migrating cell’s transendothelial electrical resistance (TER) was measured by the ECIS machine every 9 sec for up to 18 h and then analyzed. TER values at each microelectrode were pooled at discrete time points and plotted versus time [24,62]. *n* is defined as each independent microelectrode measurement for a given condition. The area under the curve (AUC) was calculated for each condition and presented as mean ± SD. Similarly, mouse VSMCs were seeded at 20,000 per well into the gold-plated electrode chamber of the ECIS assay for cellular proliferation (Applied Biophysics; 8W20idf PET). Following 72 h of starvation, the cells were treated with 10 nM of recombinant active ATX (#E-4000, Echelon Biosciences, Salt Lake City, UT, USA) with or without 10 µM of ATX inhibitor PF8380 (#B-0702, Echelon Biosciences, Salt Lake City, UT, USA) or the LPA receptor antagonist Ki16425 (#B-0730, Echelon Biosciences, Salt Lake City, UT, USA). The proliferating cell’s TER was measured by the ECIS machine every 9 sec for up to 48 h and then analyzed [62]. TER values at each microelectrode were pooled at discrete time points and plotted versus time. *n* is defined as each independent microelectrode measurement for a given condition. The area under the curve (AUC) was calculated for each condition and presented as mean ± SD.

### 4.5. Hematoxylin and Eosin Staining

Following carotid artery harvest, it was fixed in 10% paraformaldehyde for 24 h at room temperature. The next day, the tissue underwent dehydration in graded ethanol, was cleared with xylene, was embedded in paraffin, and then cut into 5 µm sections. Next, the sections were deparaffinized in xylene and rehydrated in a graded ethanol series. Next, the sections were stained with hematoxylin for 5 min and eosin for 3 min at room temperature. Images of carotid artery sections in the different groups were taken using a light microscope with a CellSens Entry 1.9 imaging system (Olympus Corporation, Tokyo, Japan). The intimal, media, and lumen areas were calculated based on the hematoxylin and eosin staining.

### 4.6. Wound Healing Assay (Scratch Assay)

A wound healing assay was conducted to measure cell migration [18,34,35]. First, cells were grown to confluence in 6-well plates, serum starved for 72 h, and a straight wound was made using a sterile 200 μL pipette tip. Serum-free media with 10 nmol/L of recombinant active ATX (#E-4000, Echelon Biosciences Inc., Logan, UT, USA), 10 μmol/L 18:1 LPA (#857230P, Avanti Polar Lipids, Birmingham, AL, USA), and 10 μmol/L ATX inhibitor (PF-8380; Echelon Biosciences, B-0702, Salt Lake City, UT, USA) or 10 µM of LPA receptor antagonist Ki16425 (Echelon Biosciences, B-0730, Salt Lake City, UT, USA) were added to the cells. As the control for LPA, LPC, and PF-8380, the same amount of 0.1% delipidated FBS (#S5394, Sigma-Aldrich, St. Louis, MO, USA) was used for the dilutions and was added to the control wells. The straight wound was photographed and measured under a microscope at 0 h and 16 h using the Nikon brightfield microscope. 

### 4.7. Western Blot Analysis

Mouse VSMCs were treated with control, active ATX, or active ATX with an inhibitor and washed with PBS following treatments. The cell lysate was prepared in ice-cold RIPA lysis buffer (#89900, ThermoFisher Scientific, Waltham, MA, USA). The cell lysate was centrifuged at 12,000× *g* at 4 °C for 15 min to obtain the protein estimation supernatant. SDS-PAGE was performed with 40 µg of total protein per well. After SDS-PAGE, the proteins were transferred to a PVDF membrane and visualized using an LI-COR Odyssey (LI-COR Biosciences, Lincoln, NE, USA).

### 4.8. Immunofluorescence Staining for Cell Proliferation

For immunofluorescence staining, mouse VSMCs were cultured on chambered slides. After incubating with 10 nM of active ATX media or no active ATX media, the cells were fixed with 4% paraformaldehyde and permeabilized using 0.2% Triton X-100. The cells were blocked with 10% goat serum for 1 h and then incubated overnight at 4 °C with mouse anti-PCNA (#13-3900, Invitrogen, Waltham, MA, USA). Next, the cells were incubated with AlexaFluor 488 goat anti-mouse (#A28175, ThermoFisher Scientific, Waltham, MA, USA) for 1 h at room temperature. The cells were mounted with a Vecta shield mounting medium with DAPI (#H-1500, Vector Laboratories, Newark, CA, USA) and visualized using a fluorescence microscope with a mounted Nikon camera.

### 4.9. Immunohistochemistry

The paraffin-embedded sections were deparaffinized and rehydrated, which was followed by antigen retrieval in boiling citrate buffer for 30 min. The sections were surrounded with a hydrophobic pen and rinsed with PBS. The sections were then permeabilized with 0.1% Triton X100 for 5 min in goat serum. The sections were blocked with 10% goat serum for 1 h and then incubated overnight at 4 °C with mouse anti-ATX (ab140915, Abcam, Waltham, MA, USA), mouse anti-lysophosphatidic acid (#504B3, Echelon Biosciences Inc., Logan, UT, USA), anti-phospho-ERK (#9101, Cell Signaling Technology, Inc. Waltham, MA, USA), PCNA (#13-3900, Invitrogen, Waltham, MA, USA), and anti-smooth muscle actin (#ab5694, Abcam, Waltham, MA, USA) as primary antibodies. The sections were then incubated with AlexaFluor 488 goat anti-mouse (#A28175, ThermoFisher Scientific, Waltham, MA, USA) and AlexaFlour 594 goat anti-rabbit (#A11012, ThermoFisher Scientific, Waltham, MA, USA) before then being rinsed with PBS. After 1 h, the sections were rinsed with PBS three times, mounted with a vector-shield mounting medium with DAPI (#H-1500, Vector Laboratories, Newark, CA, USA), and visualized using a Leica confocal microscope. 

### 4.10. Real-Time PCR mRNA Expression of LPA Receptors

The TaqMan gene expression assay and TaqMan Universal PCR Master Mix (4444556, Applied Biosystems, Foster City, CA, USA) RT-PCR reaction kit were used on an ABI Viia 7 real-time PCR system (Applied Biosystems, Foster City, CA, USA). The delta CT method normalized to glyceraldehyde-3-phosphate dehydrogenase (GAPDH) was used to analyze receptor expression. The change in expression was calculated using the 2^-ΔΔCT^ method normalized to GAPDH as an endogenous control. The primers used were Mm99999064_m1 (*IL6*), Mm01200332_g1 (*Enpp2*), Mm01346925_m1 (*LPAR1*), Mm00469562_m1 (*LPAR2*), Mm00469694_m1 (*LPAR3*), Mm02620784_s1 (*LPAR4*), Mm02621109_s1 (*LPAR5*), Mm00725412_s1 (*Acta2*), Mm00487032_m1 (*Cnn1*), Mm00441661_g1 (*Tagln*), Mm00443013_m1 (*Myh11*), and Mm99999915_g1 (*GAPDH*), which were obtained from Applied Biosystems (Foster City, CA, USA).

### 4.11. Superoxide Measurement

A high-performance liquid chromatography (HPLC) system coupled with UV-vis and fluorescence detectors was used for the measurement of superoxide in carotid tissue [25]. For mouse carotid tissue, 0.3 mg DHE (DHE-5-ethyl-5, 6-dihydro-6-phenyl-3, 8- diaminophenanthridine; Fluka, 37291) was prepared in DMSO and administered intraperitoneally 1 h before the completion of tissue isolation. Vessels were collected after reperfusion and homogenized in 50 mM phosphate buffer (pH 7.4). The supernatant was separated, and 100 μL was transferred to a tube with 100 μL of MeOH/HClO4 solution. Protein was precipitated by incubating the tube on ice for 2 h. The supernatant was obtained by centrifuging the tubes at 12,000× *g* at 4 °C for 10 min. An aliquot of 100 μL of supernatant was added to 100 μL of 1 M phosphate buffer (pH 2.6). Excess buffer was removed following centrifugation at 12,000× *g* at 4 °C for 5 min. The supernatant was transferred to a new tube for HPLC superoxide measurement [24,63].

### 4.12. Statistical Analysis

Statistical analysis was performed using Prism 8.0 (GraphPad Prism, version 8.4.2, San Diego, CA, USA, RRID: SCR_002798). Data are reported as means ± standard deviation (SD), with all experiments carried out with a minimum of three replicates. One-way analysis of variance (ANOVA) followed by the Bonferroni post hoc test or the unpaired Student’s *t*-test (Mann–Whitney U test: non-parametric t-test to compare whether there is a difference in the dependent variable for two independent groups) was used to identify significant differences between groups. A *p*-value of less than 0.05 was considered significant.

## 5. Conclusions

Emerging evidence supports LPA’s role in regulating vascular development and function. Our data add to the weight of evidence suggesting that the ATX-LPA signaling nexus may be significant in regulating vascular vessel remodeling in adult animals. We propose that these observations reflect the role ATX performs in enhancing local signaling by lysophospholipid mediators. Our observations suggest that novel strategies to inhibit the ATX-LPA axis could be therapeutically targeted in restenosis to minimize neointimal hyperplasia. 

## Data Availability

Not applicable.
